# Severe electrolyte derangements from lysozymuria in acute myeloid leukemia

**DOI:** 10.1002/jha2.464

**Published:** 2022-05-16

**Authors:** Juliana Pérez‐Pinzón, Rebecca L. Olin, Rahul Banerjee

**Affiliations:** ^1^ School of Medicine Universidad de los Andes Bogotá DC Colombia; ^2^ Department of Medicine Division of Hematology/Oncology University of California San Francisco San Francisco California USA; ^3^ Department of Medicine Division of Medical Oncology University of Washington School of Medicine Seattle Washington USA

**Keywords:** acute kidney injury, acute myeloid leukemia, hypokalemia, lysozymuria

## Abstract

Renal dysfunction in patients with acute myeloid leukemia (AML) can be multifactorial. We present the case of a 72‐year‐old male with relapsed myelomonocytic AML who presented with transient acute kidney injury (AKI) and severe persistent electrolyte derangements. In the setting of nephrotic‐range proteinuria and electrolyte wasting without significant albuminuria or glucosuria, a diagnosis of lysozymuria was made. Lysozymuria is a rare paraneoplastic complication of AML and chronic myelomonocytic leukemia characterized by lysozyme. This represents the first case of lysozymuria presenting primarily with refractory electrolyte derangements rather than severe AKI. Lysozymuria portends a poor clinical prognosis even with aggressive management.

## 1 INTRODUCTION

Renal dysfunction in acute myelogenous leukemia (AML) may occur as a result of tumor lysis syndrome, acute tubular necrosis, renal leukemic infiltration, renal hypoperfusion from sepsis, or direct nephrotoxicity from chemotherapy [1]. We report an unusual case of nephrotic‐range proteinuria and electrolyte derangements in a patient with AML with myelomonocytic differentiation. The underlying etiology was found to be lysozymuria, which to our knowledge is the first case of lysozymuria presenting with severe and refractory electrolyte derangements rather than acute kidney injury (AKI). Unfortunately, as evidenced in our case, lysozymuria portends a poor clinical prognosis even with optimal management.

## 2 CASE REPORT

A 72‐year‐old Hispanic male presented to our leukemia clinic with a 2‐week history of fatigue and anorexia. He had been diagnosed 7 months prior with AML bearing a myelomonocytic phenotype by flow cytometry. Cytogenetic analyses were normal. Molecular testing revealed FMS‐like tyrosine kinase 3 tyrosine kinase domain and isocitrate dehydrogenase 2 mutations. Prior lines of therapy included (1) idarubicin, cytarabine, and midostaurin; (2) enasidenib monotherapy; and (3) azacitidine and venetoclax beginning 3 weeks prior to his most recent presentation.

The patient's past medical and social histories were notable for hypertension and former tobacco use. He denied fevers, night sweats, or other infectious symptoms. His only medication was hydrochlorothiazide, as he had erroneously discontinued venetoclax after 1 week rather than 3 weeks. Vital signs were unremarkable, and a physical exam was notable for cachexia and gingival hyperplasia.

Relevant laboratory values included potassium 2.1 mmol/L (reference range [RR]: 3.5–5.1 mmol/L), magnesium 1.4 mmol/dl (RR: 1.8–2.4 mmol/dl), phosphate 0.6 mmol/dl (RR: 2.4–4.9 mmol/dl), and creatinine newly elevated at 1.39 mg/dl (RR: 0.6–1.24 mg/dl). White blood cell count was 31.5 × 10^9^/L (RR: 3.4–10.0 × 10^9^/L) with 44% circulating blasts. Hemoglobin was 7.9 g/dl (RR: 13.6–17.5 g/dl) and platelets 9 × 10^9^/L (RR: 140–450 × 10^9^/L). The patient's circulating blasts, anemia, and severe thrombocytopenia were similar to values 3 weeks earlier. However, his profound hypokalemia, hypomagnesemia, and hypophosphatemia with AKI were new. He was admitted for monitoring and electrolyte repletion.

Further urine testing revealed an elevated transtubular potassium gradient ratio of 16 (RR: 6–10), consistent with renal potassium wasting. Similarly, urine testing revealed an increased fractional excretion of magnesium (13.95%, RR: <2%). Nephrotic‐range proteinuria with a random urine protein‐creatinine ratio of 2.96 mg/mg (RR: <0.16 mg/mg) as well as microalbuminuria with a random albumin‐creatinine ratio of 268 mg/g (RR: <30 mg/g, cutoff >300 mg/g for macroalbuminuria) was found. However, glucosuria was detected only intermittently. Urine diuretic screening and retroperitoneal ultrasound were unremarkable.

The patient's creatinine normalized rapidly with volume resuscitation. However, electrolyte derangements remained severely refractory to high doses of intravenous repletion dosed several times per day. Fanconi syndrome was initially suspected given his recent azacitidine; however, the lack of profound glucosuria made this diagnosis less likely [2, 3]. Given the mismatch between the patient's profound nephrotic‐range proteinuria and more modest microalbuminuria, further urine testing was performed. UPEP (urine protein electrophoresis) was negative for a monoclonal spike. Serum lysozyme levels were undetectable high on two occasions (over 60 mcg/ml, RR: <11 mcg/ml).

Subsequent bone marrow biopsy revealed persistent AML with 70% myeloid blasts. Given the patient's refractory lysozymuria and persistent AML, a family meeting was held and the patient expressed his interest in returning to his home country with supportive care only. He provided permission for his case to be discussed and published. He was ultimately discharged with home hospice services and passed away 4 days after hospital discharge.

## 3 DISCUSSION

AML with myelomonocytic differentiation, formerly known as M4 AML in the French‐American‐British Classification, is rare leukemia subtyped as AML not otherwise specified in the new 2016 World Health Organization classification schema [4]. This subtype of AML is known for early leukemic infiltration, for example, gingival hyperplasia as seen in our patient [5]. In our patient's case, the mismatch between his profound proteinuria and more modest albuminuria (combined with his negative UPEP) suggested the diagnosis of lysozymuria leading to “overflow proteinuria” as the cause of his electrolyte derangements and AKI [6].

Lysozymuria remains an uncommon cause of acute renal injury. Lysozyme, also known as muramidase, is a small cationic protein with bactericidal properties that is produced by normal monocyte‐lineage cells [7]. In cases of AML with myelomonocytic or monocytic differentiation, overproduction of lysozyme can be seen [8]. Generally, free serum lysozyme is filtered out by the glomeruli before being reabsorbed by cells within the proximal convoluted tubule [9]. However, in leukemias with circulating myelocytes, this mechanism can be overwhelmed and lead to high levels of non‐albumin proteinuria from the lysozyme itself.

As shown in Table 1, lysozymuria has been reported as a complication of chronic myelomonocytic leukemia (CMML) and rarely with AML [8, 9]. Of note, only a subset of patients develop clinically meaningful AKI; regardless, electrolyte abnormalities are generally uncommon [10]. A common feature of all cases, including ours, is the poor prognosis confirmed by symptomatic lysozymuria. In patients with CMML, higher serum lysozyme levels are associated with decreased overall survival [11].

**TABLE 1:** Reported cases of lysozymuria with acute myeloid leukemia (AML) or chronic myelomonocytic leukemia (CMML)

Citation	Diagnosis	Creatinine (mg/dl)	Potassium (mmol/L)	Serum lysozyme (mcg/ml)	Urine lysozyme	Outcome
[9]	AML from antecedent CMML	2.94	4.0	NR	NR	Deceased
[12]	CMML	10.7	4.4	101	Present	Deceased
[7]	CMML	1.7	3.4	>20	NR	Deceased
[13]	AML	6.0	3.4	NR	Present	NR
[14]	AML	2.8	1.9	270	Present	Deceased
[11]	CMML (107 cases)	NR	NR	4.6–410	Present in 36% of cases	NR
[15]	AML (17 cases)	NR	2.1–4.1	1.2–348	Variable	NR
[16]	AML from antecedent CMML	NR	NR	NR	Present	NR
[17]	CMML (18 cases)	0.8–1.4	Normal in 89% of patients	5–84	Variable	NR
[18]	AML and CMML (8 cases)	NR	2.0–5.7	20–130	Variable	6/8 patients deceased
[19]	CMML	NR	NR	NR	Present	NR

Abbreviations: AML, acute myelogenous leukemia; CMML, chronic myelomonocytic leukemia; NR, not reported.

Our case is unique in multiple regards. First, our patient's creatinine rapidly recovered with fluid resuscitation while electrolyte derangements persisted. Second, the fast‐paced clinical deterioration of our patient highlights the differences between AML‐associated lysozymuria and the more insidious presentation of CMML‐associated lysozymuria. Finally, although the gold standard for diagnosis is a renal biopsy, this was not feasible in our case given our patient's AML‐related thrombocytopenia. Regardless, given his repeatedly positive serum lysozyme levels and non‐albumin proteinuria, lysozymuria sufficiently explained his clinical constellation of symptoms and laboratory abnormalities.

## 4 CONCLUSION

In conclusion, we report the first case of lysozymuria presenting with severe and refractory electrolyte derangements (and comparatively mild AKI) as a rare complication of AML with myelomonocytic differentiation. The presence of non‐albumin proteinuria and elevated serum lysozyme levels are important to make the correct diagnosis, even if the renal biopsy is unfeasible. Despite optimal clinical management with electrolyte repletion, the prognosis for patients with AML‐associated lysozymuria remains quite poor.

## FUNDING INFORMATION

The authors received no specific funding for this work.

## CONFLICT OF INTEREST

Juliana Pérez‐Pinzón reports no relevant disclosures. Rebecca L. Olin reports research funding from Cellectis; consultancy fees from Actinum. Rahul Banerjee reports research funding from Pack Health; consultancy fees from Eradigm Consulting, Guidepoint Global, Sanofi, and SparkCures; and honoraria from Curio Science.

## ETHICS STATEMENT

Consent for the publication and academic discussion of the case was obtained from the patient.
